# 

**Published:** 1869-07

**Authors:** 


### CONTENTS OF HALL'S JOURNAL OF HEALTH.

For the six months ending June, 1869.

JANUARY. Tickling in the throat. Stomach Cough. Liver cough. Consumptive cough. Nature of Croup Cough curative, and ought never to be suppressed. Premature breaking down. Good cooks. Irregular living, and its baneful effects. Throat diseases, and how brought on. Chapped hands. Cracked fingers. Nuts and cheese a good desert.

Dangerous medication. Beneficial tendencies of Young Men's Christian Associations. Sacrifice of lives. Bad Air.

February. Essence of Disease. The nature of sickness. Philosophy of all remedial means. Selfishness. How families are to grow up lovely and loving one another. Washing the face and hands. Tight boots, effects, prevention and remedy. Breathing. Cold feet. Best slippers. Health and Thrift. Ash sifters. Unhealthy houses. Sleeping habits. Fire on the hearth. Best method of heating the family sitting room.

March. The healthful influence of Bible principles in the conduct of life. Multiply and replenish the earth; the wickedness of thwarting the natural tendencies, &c. &c. A common cold; its prevention and cure. Wakefulness and how to prevent it. Common sickness. Where to live cheaply abroad: Dresden; Heidelberg; cost of living, schooling, and clothing. Sea voyages. To determine buggy sugar; loaf sugar only pure and clean. Soaps suds; "settling" water. Poisonous moulds. Bed bug riddance. The early decay among students and clergymen, and the cause.

April. Hydrophobia. Phrensy. Pulpit and Pew. Responsibilities. Mothers' influence. May moving. Spring Diseases. Stewart's poor girls' hotel; rooms and board for two dollars a week, &c.

May. Physiology of preaching; the best preachers have the best health, as witness Beecher, Hall and Spurgeon; the characteristics of each; source of renown; the value of the voice and a distinct enunciation; have a clear idea, an earnest heart. Magnetic influence. Resignation. Marrying blood relations. Changing clothing. Walking erectly. Self reliance. How to reprove. Table manners, &c.

June. Summer recreation; different kinds required by the different kinds of occupations and classes of society. The Adirondack Mountains. Going abroad. How much can be done with three month's time and five hundred paper dollars. Bronchitis, consumption and throat ail; the nature, causes and symptoms of each and how to distinguish them; the combination of symptoms which are curable, and those which are fatal; how to distinguish these diseases, &c.

July. Is the "eight hour" law a wise one? Woman's Rights; will voting add to he r happiness? will it elevate or degrade? Dyspepsia. How to eat wisely. Cold feet. Constipation. Health's three essentials. Failing eyesight. Sleeping. How to avoid taking cold for a year, 15 cts. a number.

### FOR LESS THAN NOTHING.

| | | | | | |
|---|---|---|---|---|---|
| Atlantic Monthly, . . | $4.00. | Journal of Health, . . | $1.50. | Both . | $3.90. |
| Galaxy, . . . . . | 4.00. | " " | 1.50. | Both . . | 3.90. |
| Harper's Monthly, . . | 4.00. | " " | 1.50. | Both . . | 3.90. |
| Harper's Weekly, . . | 4.00. | " " | 1.50. | Both . . | 4.00. |
| Harper's Bazar, . . | 4.00. | " " | 1.50. | Both . . | 4.00. |
| Putnam's Magazine, . | 4.00. | " " | 1.50. | Both . . | 4.00. |
| London Lancet, . . | 5.00. | " " | 1.50. | Both . . | 5.00. |
| Phrenological Journal, . | 3.00. | " " | 1.50. | Both . . | 3.00. |
| Rural New Yorker, . | 3.00. | " " | 1.50. | Both . . | 3.00. |
| Independent, . . . . | 2.50. | " " | 1.50. | Both . . | 3.00. |
| Riverside Magazine, . | 2.50. | " " | 1.50. | Both . . | 3.00. |

Any other four dollar magazine published in the United States, will be furnished for one year with Hall's Journal of Health for one year for three dollars and ninety cents; thus both publications may be had for one year for less than the price of the larger one.

Any three dollar publication in the United States, with Hall's Journal of Health, for 1869, will be furnished for $3.

If any of our present subscribers want any of the above or other publications now, they can have them on the same terms with The Journal of Health for 1870. Any of our one dollar and a-half books, or other books of same price will be sent, pp., with three copies of Hall's Journal of Health a year for $5.

### NOTICE.

In all cases let the money be sent in the form of a post-office order, payable to the order of W. W. Hall. Checks on banks or individuals for small amounts are sometimes troublesome of collection. A post-office order is perfectly safe for all parties; those who send money in letters must take the risk.

Although all the articles in Hall's Journal of Health are written by the editor himself, he feels justified in saying that in any number there is practical information as to the conduct of life in reference to health and disease which will be of more value to any one who will carry out the instructions, than fifty times the cost of a yearly subscription; there is no similar journal published in the world; its teachings are mainly derived from the editor's personal experience and observation, expressed in as few words as possible, and always plain and easily understood. Half of the ordinary diseases of life would be banished from society if the general instructions given in a few articles of the number for July, 1869, were observed.

KNEE SPRUNG HORSES permanently cured without cost or trouble. Address W. T. Baker, Sentinel Office, Waterford, N. Y.

